# Expression of concern: ‘Long non-coding RNA LINC00467 regulates hepatocellular carcinoma progression by modulating miR-9-5p/PPARA expression’ (2019), by Cai K *et al.*

**DOI:** 10.1098/rsob.230302

**Published:** 2023-08-31

**Authors:** Open Biology editorial team

*Open Biol.* **9**, 190074. (Published online 4 September 2019). (doi:10.1098/rsob.190074)

Following the publication of ‘Long non-coding RNA LINC00467 regulates hepatocellular carcinoma progression by modulating miR-9-5p/PPARA expression’, the Editorial team have concerns regarding the validity of the data used in this study.

We are issuing an expression of concern here and investigating this as a matter of urgency; we will notify readers as to the results of our investigation as soon as possible.

